# Novel SAMD9 Mutation in a Patient With Immunodeficiency, Neutropenia, Impaired Anti-CMV Response, and Severe Gastrointestinal Involvement

**DOI:** 10.3389/fimmu.2019.02194

**Published:** 2019-09-18

**Authors:** Renata Formankova, Veronika Kanderova, Marketa Rackova, Michael Svaton, Tomas Brdicka, Petr Riha, Petra Keslova, Ester Mejstrikova, Marketa Zaliova, Tomas Freiberger, Hana Grombirikova, Zuzana Zemanova, Marcela Vlkova, Filip Fencl, Ivana Copova, Jiri Bronsky, Petr Jabandziev, Petr Sedlacek, Jana Soukalova, Ondrej Zapletal, Jan Stary, Jan Trka, Tomas Kalina, Karolina Skvarova Kramarzova, Eva Hlavackova, Jiri Litzman, Eva Fronkova

**Affiliations:** ^1^Department of Paediatric Haematology and Oncology, 2nd Faculty of Medicine, Charles University and University Hospital Motol, Prague, Czechia; ^2^Institute of Molecular Genetics of the Czech Academy of Sciences, Prague, Czechia; ^3^Molecular Genetics Laboratory, Center of Cardiovascular Surgery and Transplantation, Brno, Czechia; ^4^CEITEC, Masaryk University, Brno, Czechia; ^5^Faculty of Medicine, Masaryk University, Brno, Czechia; ^6^Center of Oncocytogenetics, Institute of Clinical Biochemistry and Laboratory Diagnostics, 1st Faculty of Medicine, Charles University and General University Hospital, Prague, Czechia; ^7^Department of Clinical Immunology and Allergology, St. Anne's University Hospital Brno, Brno, Czechia; ^8^Department of Paediatrics, 2nd Faculty of Medicine, Charles University and University Hospital Motol, Prague, Czechia; ^9^Department of Paediatrics, University Hospital Brno, Brno, Czechia; ^10^Department of Medical Genetics, University Hospital Brno, Brno, Czechia; ^11^Department of Pediatric Hematology, University Hospital Brno, Brno, Czechia

**Keywords:** SAMD9, MIRAGE, immunodeficiency, neutropenia, cytomegalovirus infection, dysphagia, hematopoietic stem cell transplantation, gastrointestinal disorder

## Abstract

Mutations in the Sterile alpha motif domain containing 9 (*SAMD9*) gene have been described in patients with severe multisystem disorder, MIRAGE syndrome, but also in patients with bone marrow (BM) failure in the absence of other systemic symptoms. The role of hematopoietic stem cell transplantation (HSCT) in the management of the disease is still unclear. Here, we present a patient with a novel mutation in *SAMD9* (c.2471 G>A, p.R824Q), manifesting with prominent gastrointestinal tract involvement and immunodeficiency, but without any sign of adrenal insufficiency typical for MIRAGE syndrome. He suffered from severe CMV (cytomegalovirus) infection at 3 months of age, with a delayed development of T lymphocyte functional response against CMV, profound T cell activation, significantly reduced B lymphocyte counts and impaired lymphocyte proliferative response. Cultured T cells displayed slightly lower calcium flux and decreased survival. At the age of 6 months, he developed severe neutropenia requiring G-CSF administration, and despite only mild morphological and immunophenotypical disturbances in the BM, 78% of the BM cells showed monosomy 7 at the age of 18 months. Surprisingly, T cell proliferation after CD3 stimulation and apoptosis of the cells normalized during the follow-up, possibly reflecting the gradual development of monosomy 7. Among other prominent symptoms, he had difficulty swallowing, requiring percutaneous endoscopic gastrostomy (PEG), frequent gastrointestinal infections, and perianal erosions. He suffered from repeated infections and periodic recurring fevers with the elevation of inflammatory markers. At 26 months of age, he underwent HSCT that significantly improved hematological and immunological laboratory parameters. Nevertheless, he continued to suffer from other conditions, and subsequently, he died at day 440 post-transplant due to sepsis. Pathogenicity of this novel *SAMD9* mutation was confirmed experimentally. Expression of mutant *SAMD9* caused a significant decrease in proliferation and increase in cell death of the transfected cells.

**Conclusion:** We describe a novel *SAMD9* mutation in a patient with prominent gastrointestinal and immunological symptoms but without adrenal hypoplasia. Thus, SAMD9 mutations should be considered as cause of enteropathy in pediatric patients. The insufficient therapeutic outcome of transplantation further questions the role of HSCT in the management of patients with *SAMD9* mutations and multisystem involvement.

## Background

In 2016, Narumi et al. (1) reported mutations in Sterile alpha motif domain-containing protein 9 (SAMD9) in 11 patients examined primarily for adrenal hypoplasia. Most of the patients shared strikingly similar phenotypes, and thus, a novel multisystem disorder, MIRAGE (myelodysplasia, infection, restriction of growth, adrenal hypoplasia, genital phenotypes, and enteropathy) syndrome, was defined. Two patients from the cohort developed myelodysplastic syndrome (MDS) accompanied by loss of the chromosome 7 carrying the SAMD9 mutation. In 2017, Buonocore et al. (2) found similar *de-novo*, heterozygous *SAMD9* mutations in 8 children with a complex multisystem growth restriction phenotype. Adrenal insufficiency was frequently but not constantly present.

The appropriate treatment of the patients with SAMD9 mutations is not currently known. Fourteen of 19 patients from the first two studies died, mostly due to severe infections, in first 2 years of age. Two patients from the surviving group developed MDS with monosomy 7 and received hematopoietic stem cell transplantation (HSCT). Monosomy 7, deletions of 7q or secondary somatic loss of function mutation in SAMD9 frequently developed as a compensatory mechanism for the mutated allele, which rescued the growth-restricting effect of the *SAMD9* mutation, but it could lead to MDS in some of the patients. Schwarz reported a germline *SAMD9* mutation in three siblings with MDS and monosomy 7. Interestingly, the patients had an otherwise mild phenotype with no signs of MIRAGE syndrome except for hypospadia and bifid scrotum in one boy, and even had an asymptomatic mother carrying the same mutation (3). Bluteau et al. found 6 patients with mutated *SAMD9* and 10 patients with a mutation in SAMD9 counterpart *SAMD9L* (4) in a cohort of 86 patients with BM failure of suspected inherited origin (5). The patients presented with mild BM failure and monosomy 7, and only one presented typical signs of MIRAGE syndrome.

## Case Presentation

We describe the case of a Caucasian boy from the 4th gravidity of healthy, non-consanguineous parents. In the first month after a preterm birth (32 weeks and 3 days of pregnancy, weight 1,450 g), he manifested with bilateral bronchopneumonia and hepatopathy that progressed to septicemia with bradycardia and respiratory failure requiring ventilation support. Generalized primary cytomegalus virus (CMV) infection was confirmed at the age of 3 months. His health status was complicated by bilateral pneumonia followed by respiratory distress that demanded ventilation support complicated by disseminated intravascular coagulation and septic shock. A 6-week treatment with ganciclovir was introduced. Antimycotic treatment was introduced for suspected aspergillus infection. A huge persisting cutaneous defect in the gluteal region with uretroscrotal fistula was present from the second month of age complicated by scrotal abscess at the age of 5 months.

He suffered from recurrent upper respiratory tract infections but also sepsis of unknown origin with high fever, and high C-reactive protein (CRP) responding to antibiotic treatment. From the age of 14 months, he had recurring pneumonia with respiratory distress and septicemia at the age of 18 months. Recurrent oral, nasal and urethral candidiasis were confirmed.

### Gastrointestinal Involvement

Because of hypoproteinic malnutrition, failure to thrive and inability to swallow presumably caused by frequent vomiting, percutaneous endoscopic gastrostomy (PEG) was introduced at the age of 5 months. PEG tube management was complicated by extensive leakage. He suffered from sublingual erosions, diarrhea, recurrent proctocolitis with intestinal bleeding, and chronic perianal erosions. Hemorrhagic proctocolitis caused by *Pseudomonas aeuruginosa* with septicemia manifested at the age of 13 months. Severe *Clostridium difficile* gastroenteritis demanding intensive care manifested at the age of 23 months. Gastroscopy and colonoscopy at 18 months of age did not reveal any significant disturbances. Histologic evaluation of the duodenal mucosa showed a mild deficit of disaccharides and other enzymes of the brush-border and mild chronic non-active enteritis.

### Hematopoietic System Involvement

**Figure 1:**
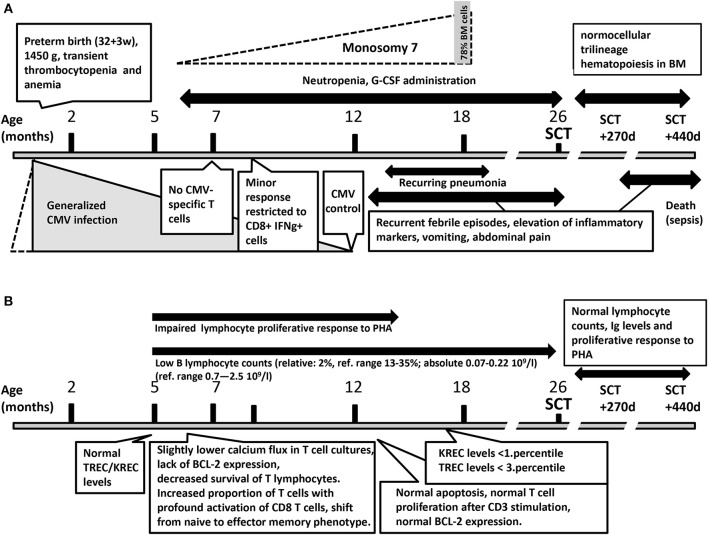
**(A)** The results of hematological testing and infectious diseases during the course of the disease. **(B)** The results of immunological testing during the course of the disease. BM, bone marrow; CMV, cytomegalovirus; IFNg, interferon gamma; SCT, hematopoietic stem cell transplantation.

Immediately after birth, the patient presented with transient thrombocytopenia and anemia (Figure 1A). From 6 months of age, he had significant neutropenia with absolute neutrophil count (ANC) <0.5 × 10^9^/l. Granulocyte colony stimulating factor G-CSF was introduced at the age of 19 months. BM evaluation at 18 months of age revealed normocellularity with reduced myeloid lineage (31%) with gradual maturation and increased erythroid lineage (48%); megakaryocytes were in the normal range, and atypical cells were not documented.

### Other Symptoms

Hypospadia, micropenis, central hypotonic syndrome, pseudobulbar syndrome, psychomotor retardation, and mild orofacial stigmatization with macroglossia and hypomimia were documented.

### HSCT

The patient was indicated for HSCT for unspecified primary immunodeficiency with severe infections, neutropenia and lack of B-cells at the age of 26 months. Conditioning regimen included busulfan targeted to plasma concentrations of 500–700 ng/mL from days−5 to−2, fludarabine 40 mg/m^2^/day from days−6 to−3 and alemtuzumab in total dose 1 mg/kg from days−8 to−6. Graft-vs.-host (GVHD) prophylaxis administered from day−1 consisted of cyclosporine A (CsA) and mycophenolate mofetil (MMF). Plasmapheresis was performed for high titers of anti-A antibodies in the situation of ABO incompatibility on days −10, −9, −8, and on day 0. He received a BM graft from his HLA identical older brother (2.6 × 10^8^ nucleated cells/kg, 5.6 × 10^6^ of CD34pos cells). Neutrophil engraftment defined as the first of 3 days with ANC above 0.5 × 10^9^/l was achieved on day +18, thrombocyte engraftment (the thrombocytes count above 20 × 10^6^/l without transfusion in previous 7 days) on day +33, respectively. Complete donor chimerism (>98% donor cells) in non-separated peripheral blood (PB) evaluated by PCR amplification of the microsatellite markers was documented from day +21. The early post-transplant period was complicated by mucositis grade III, febrile neutropenia and CMV reactivation on day +20 with good response to ganciclovir therapy. He was discharged on day +42, without signs of acute GVHD, with diarrhea, vomiting and inability to swallow, persisting from the pre-transplant period.

MMF was stopped on day +60, CsA on day +164. Recurrent febrile states with elevation of inflammatory markers, vomiting and abdominal pain started again from day +270. Gastroduodenoscopy and colonoscopy performed for suspicion of pseudo obstruction showed no pathology. In contrast with an unsatisfactory clinical condition, absolute numbers of CD3+ T cells, CD19+ B cells and CD3+CD16+56+ NK cells and proliferative response to phytohemagglutinin were comparable to controls 1 year after SCT. Serum concentrations of IgG, IgA, IgM were in the normal ranges on continuous treatment with IVIG, and BM evaluation showed normocellular trilineage hematopoiesis. On day +440, he developed sepsis with hemoculture positive for *Streptococcus salivarius* and rapid progression to septic shock and despite antibacterial treatment and intensive care he died from multiple organ failure.

## Clinical and Laboratory Investigations

### Genetic Analysis

The cytogenetic evaluation performed from PB at 5 months of age did not reveal any structural or numerical abnormality, and retrospective FISH evaluation using CEP7 probe did not reveal monosomy 7. Whole-genome SNP array and FISH analysis from BM sample taken at 18 months of age found monosomy 7 in 78.5% of interphase nuclei.

Whole-exome sequencing analysis was performed at 18 months of age. Mutations in the genes causing congenital neutropenia were excluded, as well as variants in genes causing dyskeratosis congenita, because Hoyeraal Hreidarsson syndrome was considered. No other potentially causative variants were found using the virtual panel of genes associated with bone marrow failure or immunodeficiency. After publication of the SAMD9 patient cohort in 2016, the data were reanalyzed, and a novel, previously unreported *de-novo* heterozygous mutation in the *SAMD9* gene (c.2471 G>A, p.R824Q) was reported. Although this change is predicted as tolerated by SIFT (6) and benign by PolyPhen2 (7), with a CADD (8) score of 15.2, the residue is located near previously reported mutations p.K821M (9) and p.N834Y (1), and the mutation was not found in the ExAC or gnomAD population databases (10).

### Immunological Evaluation and Functional Testing

The results of immunological testing during the course of the disease are summarized in Figure 1B. Flow cytometry (FC) determination (first performed at the age of 5 months) of major lymphocyte subsets did not provide conclusive results. CD3 cells were overrepresented; their percentages fluctuated from 87 to 96% (ref. range 39–77%), absolute CD3 cell numbers from 2.99 to 9.88 10^9^/l (ref. range 2.4–6.9 10^9^/l). CD4 cell numbers were normal: 21–42% (ref. range 25–50%), absolute number 1.34–2.28 10^9^/l (ref. range 1.04–5.10 10^9^/l). CD8+ cells were abundant: 47–69% (ref. range 13–26%), absolute number 1.62–7.49 10^9^/l (ref. range 0.6–2.2 10^9^/l). CD19+ cells were markedly decreased with repeatedly estimated representation of 2% (ref. range 13–35%), absolute number 0.07–0.22 10^9^/l (ref. range 0.7–2.5 10^9^/l). CD4/C8 ratio varied between 0.30 to 0.83 (ref 0.7–3.08) NK cell (CD16/56+CD3-) numbers were normal: from 7 to 11% (ref. range 2–13%), abs number 0.76–0.38 (ref range 0.7–1.0 10^9^/l).

Levels of immunoglobulins were elevated (IgG 10.6 g/l, IgM 7.1 g/l, IgA level was within ref. range: 0.204 g/l). Thereafter, IgG and IgA levels remained within normal levels, IgM decreased to 3.25 g/l. IgE: was repeatedly <17 UI/ml. Total hemolytic complement (CH 50) and granulocyte function test (“burst test) results were normal.

Detailed FC evaluation at 7 months of age revealed increased proportion of T cells, with profound activation of CD8 T cells (HLA-DR+ 72%) and shift from naive (6%) to effector memory phenotype (77%). This was presumably in response to persistently present CMV viremia. However, no functionally responding CMV specific T cells were detected. Minor response restricted to CD8+ IFNg+ producing cell was detected at 8.5 months of age that did not lead to CMV control. CMV reactivation control was restored only at one year of age (PCR CMV negativity). Retrospective analysis of neonatal dry blood spot did not reveal any presence of CMV.

**Figure 2:**
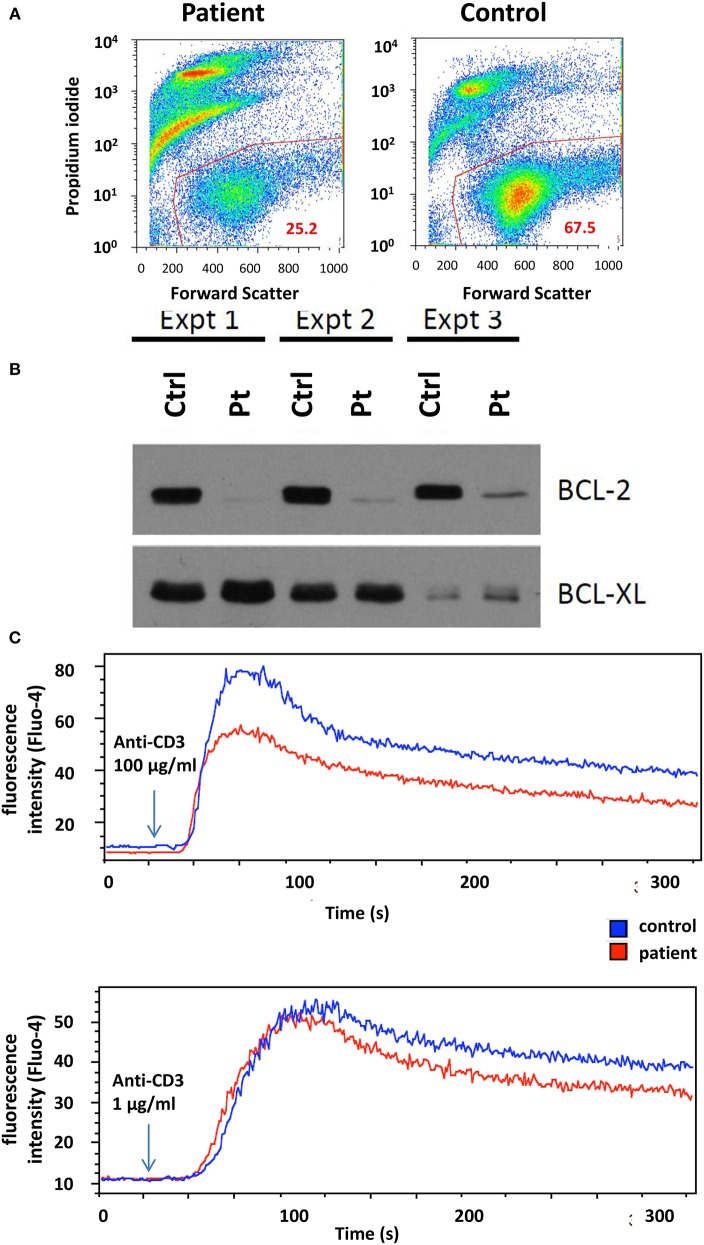
**(A)** T-cell culture viability determined by flow cytometry. Live cells were gated based on propidium iodide and forward scatter signals. **(B)** Western blot analysis of Bcl-2 and Bcl-xL expression in T cells after 2, 3, and 5 weeks of culture. Ctrl, control, Pt, patient. **(C)** Flow cytometry analysis of T-cell calcium response to activation with 1 and 100 μg/ml anti-CD3ε antibody. The arrow indicates the time point when antibody was added to the sample.

Lymphocyte proliferation in response to phytohemagglutinin (PHA) using 3H-Thymidine incorporation was repeatedly found reduced between 5 and 14 months of age. At 7 months of age, activated T-cell cultures were established by stimulating PBMCs with immobilized anti-CD3ε antibody followed by propagation in the presence of IL-2. These cultures displayed reduced viability, accompanied by the lack of anti-apoptotic Bcl-2 protein expression (Figures 2A,B). Upon CD3 re-stimulation, they showed slightly decreased calcium flux (Figure 2C), while no difference in overall tyrosine phosphorylation after TCR stimulation was observed (data not shown). However, examination of activated T-cell cultures newly established 6 months later (coincident with CMV control) revealed normal level of apoptosis, normal cell proliferation and Bcl-2 expression comparable to controls (data not shown).

Neither T-cell receptor excision (TREC) nor kappa-deleting element excision (KREC) circle levels were decreased at 5 months of age in PB, but at 17 months of age, the KREC numbers were decreased to the levels observed in SCID patients, and TREC levels were reduced below the 3rd percentile of age-matched controls.

## Functional Evaluation of *SAMD9* Mutation

**Figure 3:**
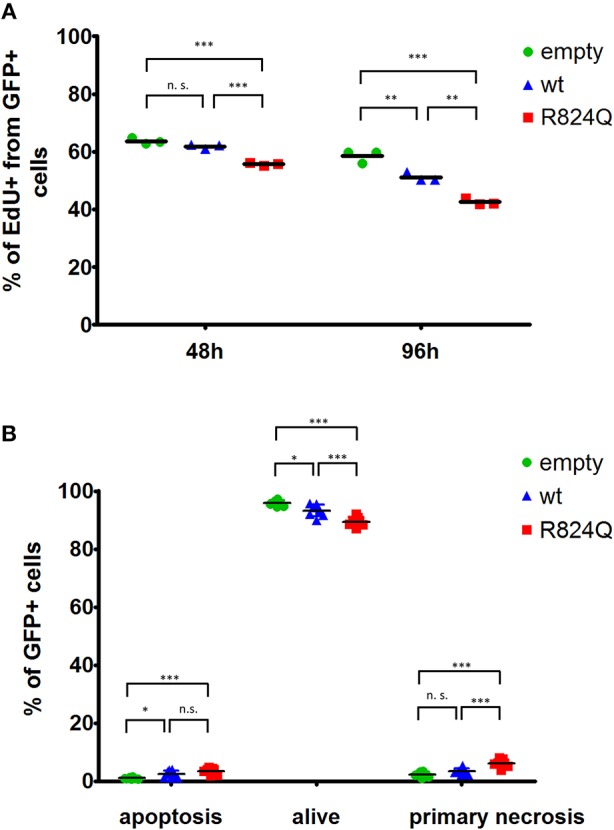
Analysis of proliferation **(A)**, cell death **(B)** of HEK293T cells transfected with wt SAMD9-GFP, mutant SAMD9-GFP or empty GFP-positive control expression vector. Representative data are shown with results depicted as a fraction of GFP+ cells. Statistical significance: (^*^P ≤ 0.05; ^**^P ≤ 0.001; ^***^P ≤ 0.0001).

Transient ectopic expression of wt SAMD9 resulted in a significant decrease in proliferation of the transfected cells. The impact of mutant SAMD9 was even more profound resulting in a dramatic drop in the number of proliferating cells (Figure 3A). Expression of SAMD9 (both wt and mutant) also caused an increase in apoptosis. Interestingly, mutant SAMD9 induced primary necrosis of the cultured cells further demonstrating the gain-of-function impact of p.R824Q on SAMD9 (Figure 3B).

## Discussion

In concordance with previously published cases (1, 2), our patient was delivered preterm and seriously ill in the neonatal period and needed intensive care. He showed genital anomalies, and he suffered from recurrent infections and chronic diarrhea. Thus, his phenotype was consistent with MIRAGE syndrome apart from the adrenal insufficiency, the sign that defined the original published cohort. Buonocore et al. reported severe adrenal insufficiency in 6 patients, while mild and no adrenal involvement were reported in the remaining two patients (2). Our patient repeatedly suffered from hyponatremia, but hyperpigmentation of the skin was not observed, and cortisol levels were only found decreased in one of three evaluations.

One of the two dominant clinical features in our patient were the inability to swallow, requiring PEG feeding, and chronic vomiting, symptoms which have not been highlighted as diagnostic signs so far. However, case presentations in the cohort of Narumi et al. reveal that at least four of 11 patients required feeding tubes, and one had a gastrostomy tube due to aspiration pneumonias, esophageal stricture, or achalasia. Also the two patients reported recently by Sarthy et al. had enteral feeding intolerance (9). Thus, SAMD9 mutations should be considered in cases of unexplained disturbances of the upper gastrointestinal tract. His other gastrointestinal symptoms included chronic diarrhea, which is a common symptom in MIRAGE syndrome [reported in 9 of 11 patients in the original cohort (1)].

The second dominant clinical feature was severe neutropenia requiring the administration of G-CSF. One of the three siblings reported by Schwartz et al. manifested with severe neutropenia, but it was associated with macrocytosis, thrombocytopenia and hypocellularity and trilineage dysplasia with 1% blasts in the BM. In our patient, BM aspiration performed at 18 months was without signs of myelodysplasia, only with reduction in the myeloid lineage. Thus, congenital neutropenia was primarily considered at that time. Monosomy 7 was revealed incidentally by SNP array, and retrospective evaluation of BM by FISH revealed monosomy 7 in the 78.5% of nuclei.

The complex immunodeficiency complications in our patient are in good accordance with manifestation of patients described by Narumi et al. where all 7 described patients were prone to complicated or recurrent infections, including episodes of pneumonia; one described patient suffered from severe CMV infection as well. In another patient, recurrent fever with high CRP was reported. Bluteau et al. reported severe recurrent infections in 4/6 patients with SAMD9 mutation (5). Despite clinically manifested immunodeficiency since birth, the laboratory immunological investigation did not show any gross abnormality. Examination of the lymphocyte subsets revealed decreased B lymphocytes, and the lymphocyte proliferation test showed decreased response after PHA stimulation. This finding is consistent with the previous observation by Narumi et al. that showed decreased numbers of B lymphocytes and decreased NK activity in several patients. Bluteau et al. reported immunoglobulin deficiency in 2/6 patients (5). The most prominent sign of immunodeficiency in our patient was non-responsiveness of T lymphocytes to CMV despite severe CMV infection in the early infancy period.

SAMD9 acts as a growth repressor, and SAMD9 mutations are considered gain-of function mutations, thus, further intensifying the growth suppression. The p.R824Q mutation present in our patient was predicted as benign by two prediction tools. However, neither PolyPhen2 (7) nor SIFT (6) tools are adjusted for gain-of-function predictions and, thus, should be used with caution (11). Growth of HEK293 cells transfected with SAMD9 mutants was profoundly restricted in several studies (1, 9, 12). The cells transfected with the p.R824Q mutant showed a significant growth restriction as well. Interestingly, we also observed an increase in cell death of the cultured cells. The involvement of SAMD9 in cell death has already been predicted (13). During the initial evaluation of our patient, we observed a reduced growth, higher rate of apoptosis of cultured T lymphocytes together with the lack of Bcl-2 expression. However, this observation was not confirmed during repeated evaluation after 6 months. We can speculate that the renewal of the proliferation capacity could have been caused by the gradual emergence of cells with compensatory loss of chromosome 7, as observed in other studies (2).

The role of HSCT in the management of patients with SAMD9 mutations is not entirely clear. To our knowledge, 14 transplanted patients with SAMD9 mutation have been reported so far, including our patient. One patient from the cohort of Narumi et al. (1) was transplanted due to MDS, but died due to Epstein-Barr virus-related post-transplant lymphoproliferative disorder. The only 2 surviving patients from the cohort described by Buonocore et al. (2) were also transplanted due to MDS. However, these patients had the mildest phenotype, with only fewer syndromic features, compared to the rest of the patients. This was also the case of three other reported patients (3, 14) who survived after HSCT. Wilson et al. retrospectively identified a SAMD9 mutation in a patient with MIRAGE phenotype after HSCT due to MDS. The patient survived more than 10 years after transplant but suffered from multiple other medical issues related to syndromic features (12). Bluteau et al. reported four transplanted patients, of whom three survived without major complication, while the only one patient with MIRAGE phenotype died. Interestingly, 11 of 13 patients with SAMD9 or SAMD9L mutations, who were not transplanted immediately, showed spontaneous improvement in blood cell counts, and HSCT was even canceled in 5 of them with no impact on survival (5). Recently, Sarthy et al. reported two patients with SAMD9 mutations and severe MIRAGE phenotype transplanted due to BM failure, who both died after HSCT due to multiorgan complications connected with the syndrome (9). This was also the case of our patient, who tolerated relatively well the transplantation procedure and successfully restored hematopoiesis, but died 14 months after HSCT due to worsening of his other symptoms. Taken together, 5 of 6 reported patients with the MIRAGE phenotype died after SCT, while all 9 reported patients without severe syndromic features survived. These results show that in patients with the MIRAGE phenotype, the transplantation management is rarely successful due to accompanying multiorgan issues. BM disturbances can show spontaneous improvement, including the disappearance of monosomy 7. Thus, a watch and wait strategy should be an option for both syndromic and non-syndromic patients. However, their treatment must be managed by primary immunodeficiency centers with access to intensive care units with multidisciplinary teams.

In conclusion, we report the case of a patient with novel mutation in *SAMD9* with severe gastrointestinal involvement, neutropenia and immunodeficiency, underscoring the role of *SAMD9* in the differential diagnosis of patients with these symptoms. We further question the role of HSCT in the management of the disease.

## Methods

The detailed descriptions of CMV response detection (15), activated T-cell culture (16), cell viability, BCL-2 expression, calcium response (16), TREC/KREC analysis, and of the functional assessment of SAMD9 mutation *in-vitro* are available in the Supplementary File (17–19).

## Ethics Statement

This study was carried out in accordance with the recommendations of the 2nd Medical Faculty Ethics Committee Guidelines. The parents of the patient gave written informed consent with the study in accordance with the Declaration of Helsinki. The protocol was approved by the Ethics Committee of the 2nd Medical Faculty, Charles University Prague. Written informed consent was obtained from the parents of the participant for the publication of this case report.

## Author Contributions

RF, EF, TK, VK, MS, and TB analysis and interpretation of data for the study and drafting the manuscript. MR, KS, VK, MS, TB, EH, and HG functional experiments. PR, MZ, EM, TF, ZZ, EH, JB, PJ, PS, JSo, JSt, MV, JL, JT, IC, FF, PK, and OZ analysis and interpretation of data.

### Conflict of Interest Statement

The authors declare that the research was conducted in the absence of any commercial or financial relationships that could be construed as a potential conflict of interest.
