# Supplementary material for: Novel SAMD9 Mutation in a Patient With Immunodeficiency, Neutropenia, Impaired Anti-CMV Response, and Severe Gastrointestinal Involvement
Source: Front Immunol. 2019 Sep 18;10:2194. doi: 10.3389/fimmu.2019.02194 (PMC6759462; doi:10.3389/fimmu.2019.02194)
Supplement: Supplementary file 1 [file Data_Sheet_1.pdf]

Novel SAMD9 mutation in a patient with immunodeficiency, neutropenia, impaired anti-CMV response and severe gastrointestinal involvement

Supplementary Methods

CMV response

CMV-specific T cell-response was detected by intracellular cytokine staining using flow cytometry after *ex vivo* stimulation with CMV antigen as described in detail previously.[1]

Activated T cell culture

Peripheral blood mononuclear cells (PBMC) prepared after Ficoll-Paque density centrifugation of 1 ml blood were activated by immobilized anti-CD3ε antibody MEM-57[2] in the presence of 50 μ/ml IL-2 (Proleukin, Novartis) in RPMI/FCS. After 2 days, the cells were removed from the antibody and propagated in RPMI/FCS and 50 μ/ml IL-2.

Cell viability analysis

Cells were stained with 0.5 μg/ml propidium iodide (Thermo Fisher Scientific) in PBS and directly analyzed by flow cytometry.

BCL-2 expression

In total, 5×10^6 cells were lysed in 0.2 ml SDS-PAGE sample buffer. The lysates were sonicated for 10 s, and Bcl-2 and Bcl-XL content was analyzed by immunoblotting with respective antibodies (Cell Signaling Technology).

Calcium response

In total, 5×10^6 cells were loaded with 1 μg Fluo-4 calcium sensing dye (Molecular Probes, Thermo Fisher Scientific) in 0.5 ml RPMI/FCS for 30 min at 37°C. Cells were washed, stimulated with varying concentrations of MEM-92 (IgM antibody to CD3ε[3]) and the florescence was continuously measured by flow cytometry for 5 min.

The analysis of T-cell receptor excision circles (TRECs) and kappa-deleting excision circles (KRECs)

PCR-based quantification of TREC and KREC levels from the PB was performed as described previously.[4,5]

Functional assessment of *SAMD9* mutation

The c.2471 G>A mutation was introduced into pCMV6-AC-GFP vector expressing GFP-tagged wild-type *SAMD9* cDNA (RG219076; Origine) by site-directed mutagenesis. Mycoplasma-

negative HEK293T cells (DSMZ) were transfected with wt SAMD9-GFP, mutant SAMD9-GFP or empty GFP-positive control expression vector using polyethylenimine. The GFP+ cells were analyzed by proliferation (after 48 and 96 hours) or apoptosis assay (after 96 hours). Experiments were performed in two biological replicates.

Proliferation assay

Transfected HEK293T cells were cultured for 1.5 hours with 10 µM EdU (Click-iT® Plus EdU Flow Cytometry Kit, Thermo Fisher Scientific), harvested, spun down, and fixed using 10% formaldehyde (Thermo Fisher Scientific) for 10 min at room temperature. Fixed cells were permeabilized using 0.1% Triton X-100 (Thermo Fisher Scientific) for 15 min at 37°C, washed with 1% bovine serum albumin in PBS, spun down and stained with Alexa Fluor 647 picolyl azide for 30 min at room temperature according to manufacturer's protocol. Finally, staining with DNA intercalator 4′,6-diamidino-2-phenylindole (DAPI, Thermo Fisher Scientific) was performed for 30 min at 4°C, the cell were washed with PBS and measured on BD LSRII flow cytometer. EdU positivity was determined using FlowJo software (BD Biosciences).

Apoptosis assay

Transfected HEK293T cells were harvested, spun down and resuspended in Annexin V Binding Buffer (Exbio). The cells were stained with Annexin V-Dyomics 647 (Exbio) and DAPI for 30 minutes in the dark on ice. The samples were washed with Annexin V Binding Buffer and measured on BD FACS Celesta flow cytometer. Fractions of Annexin V+ apoptotic cells (Annexin V+ DAPI- (early apoptosis) and Annexin V+ DAPI+ (late apoptosis/secondary necrosis)), and Annexin V- DAPI+ primary necrotic cells were determined using FlowJo software (BD Biosciences).

Statistical analysis

Data from proliferation and apoptosis assays were analyzed using Prism 7 (GraphPad Software, La Jolla, USA). Differences between wt SAMD9, mutant SAMD9 and control cells were compared using 1-way ANOVA with Bonferoni´s multiple comparison posttest.

References

1. Pelák O, Stuchlý J, Król L, et al. Appearance of cytomegalovirus-specific T-cells predicts fast resolution of viremia post hematopoietic stem cell transplantation. *Cytom. Part B Clin. Cytom.* 2017;92(5):380–388.

2. Horejsí V, Angelisová P, Bazil V, et al. Monoclonal antibodies against human leucocyte antigens. II. Antibodies against CD45 (T200), CD3 (T3), CD43, CD10 (CALLA), transferrin

receptor (T9), a novel broadly expressed 18-kDa antigen (MEM-43) and a novel antigen of restricted expression (MEM-74). *Folia Biol. (Praha).* 1988;34(1):23–34.

3. Knapp W. Leucocyte typing IV : white cell differentiation antigens. New York: Oxford University Press; 1989.

4. Sottini A, Ghidini C, Zanotti C, et al. Simultaneous quantification of recent thymic T-cell and bone marrow B-cell emigrants in patients with primary immunodeficiency undergone to stem cell transplantation. *Clin. Immunol.* 2010;136(2):217–27.

5. Froňková E, Klocperk A, Svatoň M, et al. The TREC/KREC assay for the diagnosis and monitoring of patients with DiGeorge syndrome. *PLoS One*. 2014;9(12): e114514
